# Hospital Meetings

**Published:** 1907-03-30

**Authors:** 


### HOSPITAL MEETINGS.

### UNIVERSITY COLLEGE HOSPITAL.

The annual general meeting of members of the Corporation of University College Hospital took place on Friday afternoon, the 22nd instant, Mr. Henry Lucas, Chairman of the General Committee, in the chair.

The President, the Duke of Bedford, was re-elected. Sir Donald Currie, through whose generosity the Medical School is in course of erection, was added to the number of vice-presidents. Mr. Henry Lucas was elected treasurer, and, while accepting the position, he reminded the meeting that he would only fulfil that duty until a substitute might be found, as he did not consider it advisable that he should hold both the treasurership and the chairmanship of the General Committee.

The Chairman, in reviewing the salient features of the report for 1906, referred to the opening of the new hospital in November last by the Duke of Connaught, who had since consented to be one of their vice-patrons. The hospital was incorporated on January 1, and had become responsible for the Medical School, the work of which was being continued at University College until the new buildings were completed, which they hoped would be in the course of the summer. The Home for Nurses and the Students' House would also, it was hoped, be then ready for occupation. They had again to thank the Ladies' Association for showing their deep interest in the work of the hospital by arranging for the maintenance of a cot, in addition to the beds already maintained by the association.

The Chairman said he much regretted to have to point out that the subscriptions had decreased, for though the donations had considerably increased, they were an uncertain source of income. The financial condition was serious, as the necessary expenditure was nearly £28,000 per annum, and the reliable income was only £9,000, and the committee had since January 1906 been obliged to sell out stock to the value of £12,286, notwithstanding which the year closed with a debt of £8,600.

The committee regretted that, owing to his many engagements, Sir Victor Horsley had felt obliged to tender his resignation from the active staff, but they were glad he was remaining with them as consulting surgeon.

The meeting terminated with a vote of thanks to the Chairman.

### METROPOLITAN CONVALESCENT INSTITUTION.

The annual meeting of the Metropolitan Convalescent Institution was held on March 20, Mr. M. O. Fitzgerald, the Chairman of the Board, presiding. The report for 1906 records a year's work greater in extent than any that has preceded it. The total number of patients admitted to the homes at Walton, Broadstairs, and Bexhill was 7,553—nearly 500 in excess of any previous year. Attention was called to the satisfactory working of the arrangement for the reception at Walton of a limited number of patients suffering from pulmonary consumption in the early stage of the disease, and to the fact that in order to extend the usefulness of the institution accommodation had recently been provided in special wards at the Bexhill Homes for surgical cases requiring treatment. The income of the charity for general purposes in 1906 was £14,183 1s. 11d., and the expenditure amounted to £14,748 10s. The Board appeals for £7,000 required for the completion of the Men's Home at Little Common, Bexhill, which was opened with seventy-one beds in 1905.

